# Characteristics and Treatment Patterns of Patients with Haemophilia B Receiving Recombinant Coagulation Factor IX

**DOI:** 10.3390/jcm14134555

**Published:** 2025-06-26

**Authors:** Young-Shil Park, Tai Ju Hwang, Sang Kyu Park, Eun Jin Choi, Jeong A Park, Hee Jo Baek, Chuhl Joo Lyu, Jae Hee Lee, Mi Kyung Kim, Ji Yoon Kim, Sun Ah Lee, Boram Park, Da-Hye Kim, Sung Beom Chung, Chung-Mo Nam, Yaeji Lee, Ki Young Yoo

**Affiliations:** 1 Department of Pediatrics, Kyung Hee University Hospital at Gangdong, Seoul 05278, Republic of Korea; pysped@khu.ac.kr; 2 Korea Hemophilia Foundation Clinic, Gwangju 61932, Republic of Korea; tjhwang@chonnam.ac.kr; 3 Korea Hemophilia Foundation Clinic, Busan 47012, Republic of Korea; sangp1@nate.com; 4 Department of Pediatrics, Daegu Catholic University Medical Center, Daegu 42472, Republic of Korea; ejchoi2@cu.ac.kr; 5 Department of Pediatrics, Inha University Hospital, Incheon 22332, Republic of Korea; parkj@inha.ac.kr; 6 Department of Pediatrics, Chonnam National University Hwasun Hospital, Chonnam National University Medical School, Jeollanam-do 58128, Republic of Korea; swan93@naver.com; 7 Department of Pediatric Hematology Oncology, Yonsei University College of Medicine, Seoul 03722, Republic of Korea; cj@yuhs.ac; 8 Department of Pediatrics, Chungbuk National University Hospital, Cheongju 28644, Republic of Korea; pedjhl@gmail.com; 9 Department of Pediatrics, Presbyterian Medical Center, 365 Seowon-ro, Wansan-gu, Jeonju 54987, Republic of Korea; kmik7@hanmail.net; 10 Department of Pediatrics, School of Medicine, Kyungpook National University, Kyungpook National University Hospital, Daegu 41944, Republic of Korea; phojyk@gmail.com; 11 Department of Internal Medicine, Daegu Fatima Hospital, Daegu 41199, Republic of Korea; mozarteffect@hanmail.net; 12 Medical Department, Pfizer Pharmaceuticals Korea Ltd., Seoul 04631, Republic of Korea; bo-ram.park@pfizer.com (B.P.); da-hye.kim@pfizer.com (D.-H.K.); sung-beom.chung@pfizer.com (S.B.C.); 13 Department of Preventive Medicine, Yonsei University College of Medicine, Seoul 03722, Republic of Korea; cmnam@yuhs.ac; 14 Department of Biostatistics and Computing, Yonsei University, Seoul 03722, Republic of Korea; ysbiostat@yuhs.ac; 15 Korea Hemophilia Foundation Clinic, Seoul 06641, Republic of Korea

**Keywords:** haemophilia B, factor IX, treatment adherence and compliance, annual bleeding events, prophylactic treatment, real-world evidence

## Abstract

**Introduction:** In Haemophilia B, guideline-level factor IX (FIX) prophylaxis is recommended, but real-world dosing and adherence vary. **Aim:** To assess treatment patterns, adherence, FIX dosing, and their associations with bleeding events in Korean patients. **Methods:** We conducted a retrospective chart review and one-time survey of 130 Korean patients with haemophilia B treated with FIX for ≥12 months at 12 centers (June 2022–May 2023). A total of forty-seven patients (36.2%) received prophylaxis (≥90 IU/kg/week for ≥45 weeks); the remainder were managed non-prophylactically. Annualized bleeding events (ABEs) were analyzed using negative binomial regression, and monthly bleeds with a generalized linear mixed model. Covariates with *p* < 0.10 and clinical relevance were included in multivariable models. **Results:** The prophylaxis group showed significantly fewer ABEs (incidence rate ratio [IRR]: 0.383, *p* = 0.011). Each 100 IU/kg monthly dose increment reduced bleed risk (IRR: 0.692, *p* < 0.001). Adherence showed no independent association with bleeding in adjusted models. **Conclusions:** Bleed prevention in haemophilia B is driven more by delivered FIX exposure than by regimen label. Study-defined sustained prophylaxis remains underused and under-dosed. Individualized dosing and continuous adherence monitoring are essential to close this treatment gap and improve outcomes.

## 1. Introduction

Haemophilia B is an X-linked bleeding disorder characterized by the insufficient activity of coagulation factor IX (FIX) clotting activity, which predisposes patients to delayed haemostasis and recurrent haemorrhage after minor trauma or medical procedures [1,2]. Intravenous FIX concentrates—initially plasma-derived and now largely recombinant standard-half-life (SHL) formulations—have underpinned both on-demand therapy and routine prophylaxis for more than two decades [3]. Although extended-half-life (EHL) concentrates, gene transfer, and subcutaneous non-factor agents have recently entered clinical practice, their uptake remains limited by age restrictions, anti-vector immunity, monitoring requirements, and cost [4,5]. Consequently, conventional FIX replacement remains the mainstay for most children, peri-operative cases, and many adults worldwide [6,7,8]. International guidelines released by the World Federation of Haemophilia (2020) [6] and the International Society on Thrombosis and Haemostasis (2024) [8] continue to designate FIX-based prophylaxis as first-line care for severe haemophilia B, citing consistent evidence that early and sustained prophylaxis not only reduces bleeding frequency but also substantially delays the onset of joint damage—a finding particularly relevant in pediatric and adolescent populations [5,9].

A clinical benefit, however, hinges on sustained adherence. Self-reported compliance with prophylaxis varies widely (30–90%) across age groups [10,11,12]; lapses are driven by asymptomatic periods, treatment fatigue, venous-access issues, and socioeconomic constraints [10,13,14]. Simplified schedules, such as once-weekly dosing at (100 IU/kg), have improved convenience in several trials [1,15], yet regimen selection must ultimately reflect individual pharmacokinetics, lifestyle, and preferences.

Asian real-world data on how dosing intensity and adherence translate into bleeding control remain limited. In Korea, most patients with haemophilia B continue to use SHL FIX products, but dosing practices vary considerably. To date, no nationwide chart-based Korean study has evaluated the impact of real-world dosing patterns and adherence on annualized bleeding events (ABEs).

To address this evidence gap, the present study aimed to systematically evaluate treatment practice and outcomes among Korean with haemophilia B. Specifically, this multi-center chart review and patient survey therefore aimed to (i) characterize current FIX utilization, (ii) quantify adherence, and (iii) assess the association between regimen type, cumulative FIX exposure, and ABEs in Koreans with haemophilia B. These real-world findings seek to inform practical strategies that reconcile global recommendations with domestic clinical realities.

## 2. Materials and Methods

### 2.1. Study Design

This study employed a hybrid design combining a retrospective chart review and a cross-sectional patient survey conducted from June 2022 to May 2023 at three Korea Haemophilia Foundation clinics and nine tertiary hospitals, which are representative of haemophilia treatment centers in Korea. Patients diagnosed with haemophilia B (or their parent or legal representative who administered FIX to patients with haemophilia B), treated with FIX for at least 1 year and who signed an informed consent document were included in the study. However, patients who had participated in an interventional study within the past year or were treated with a bypassing agent were excluded. The study design was approved by the Institutional Review Board of all participating institutions and all participants voluntarily signed the informed consent document.

### 2.2. Data Sources

Data were collected through medical chart reviews and patient surveys. The medical chart review included patient demographics, clinical characteristics, treatment patterns, and bleeding events over the past year. Treatment patterns included duration, regimens, dosage, and frequency. The patient survey focused on patients’ adherence to treatment, reasons for non-adherence, and any bleeding events within 1 month after the survey.

Patients were categorized based on the medical chart review over the past year. Those who received prophylaxis at a dose of 90 IU/kg/week or more for over 45 weeks, as defined by a Canadian multi-center study [16], were included in the prophylactic treatment group. However, patients who did not meet these criteria were included in the non-prophylactic treatment group.

### 2.3. Outcome Measures

The primary endpoint was the number of ABEs per patient. An ABE was defined as the total number of bleeding episodes documented in the medical record during the 12-month observation window (June 2022 to May 2023). Since all participants contributed a full year of follow-up, this measure reflects a direct count rather than an extrapolated estimate based on partial observations.

The secondary endpoint was the monthly bleeding events derived by dividing each patient’s annual data into calendar months. This allowed the use of a repeated-measures framework to assess time-varying effects.

Each bleeding episode was further categorized as mild, moderate, or severe based on the clinical urgency and requirement for clotting factor replacement. Mild bleeding did not require clotting factor treatment; moderate bleeding necessitated clotting factor replacement but was not life-threatening; and severe bleeding required immediate clotting factor administration to prevent life-threatening outcomes or serious disability.

In this study, the term “annualized bleeding events” was used instead of the more common “annualized bleeding rate (ABR)” to more accurately reflect the nature of the data. As every patient had a complete 12-month follow-up period, reporting the absolute number of events was considered more transparent and appropriate than implying a rate derived from varying observation durations, as is often the case in other studies.

### 2.4. Statistical Analysis

The study aimed to investigate the relationship between ABEs and various demographics, clinical characteristics, treatment patterns, and patient adherence. Spearman’s correlation analysis was used to assess the correlation between ABEs and continuous variables, while Mann–Whitney *U* or Kruskal–Wallis tests were used to compare ABEs between two or more groups.

To further explore the associations between ABEs and treatment patterns as well as patient adherence, two statistical models were applied. First, negative binomial regression was conducted with the total number of bleeding events per year as the response variable. Second, a generalized linear mixed model with a negative binomial distribution was applied, considering repeated time as a random variable and the number of bleeding events per month as the response variable. The results were reported as the coefficient, standard error, exponentiated estimates, and 95% confidence interval. Covariates included age, sex, body mass index, height, weight, treatment duration, disease severity, arthropathy, human immunodeficiency virus status, hepatitis B status, FIX activity level, half-life, total factor consumption, injection administrator, treatment regimen switch, and bleeding history in the past month. Covariates with a *p*-value less than 0.1 in univariate analysis and clinically relevant covariates were included in multiple regression analyses for both statistical models. All statistical analyses were conducted using SAS software version 9.4 (SAS Institute Inc., Cary, NC, USA) and R software version 4.3.0 (R Foundation for Statistical Computing, Vienna, Austria; http://www.R-project.org (accessed on 1 August 2023)).

## 3. Results

### 3.1. Patients’ Characteristics and Treatment Patterns

A total of 130 patients were included in the study, with a mean age of 31.56 ± 16.99 years and a mean treatment duration of 241.20 ± 116.43 months. Table 1 summarizes the baseline demographics, clinical characteristics, and treatment patterns of patients based on a 1-year medical chart review. Patients were divided into a prophylactic treatment group (47 patients; 36.15%) and a non-prophylactic treatment group (83 patients; 63.85%).

The average prescribed prophylaxis dose was 86.26 ± 26.84 IU/kg/week, administered 1.56 ± 0.61, times per week. No patient switched between standard half-life and extended half-life (EHL) over 1 year.

Dose adjustments were frequent: 99 patients (78.57%) experienced at least one change during the year. More than 90% of these changes were attributed to differences in the actual potency of each vial rather than clinical reasons. The subacute stage after surgery was observed in 4.02% of patients and a lack of preventive effect was reported in 2.97% of patients. Full details on the frequency and reasons for dose modification are given in Supplementary Table S1.

### 3.2. Prophylacxis Uptake and FIX Exposure

In total, 47 of 130 patients (36.15%) met the predefined prophylaxis criterion (≥90 IU/kg/week for ≥45 weeks). As shown in Table 2A, uptake was highest in adolescents (69.23%) and lowest in adults (31.37%). Prophylaxis frequency also rose with disease severity, rising from 0% in mild cases and 20% in moderate cases to approximately 41% in patients classified as severe. Weekly FIX exposure differed not only between treatment groups but also across severity strata (Table 2B). In the prophylaxis group, severe and moderate patients received similar doses (106.78 IU/kg/week and 104.38 IU/kg/week, respectively), whereas non-prophylaxis patients with severe disease received substantially less FIX (74.06 IU/kg/week). Factor consumption patterns mirrored these findings. Detailed subgroup values, including EHL versus SHL products and full *p*-values, are provided in Supplementary Table S2.

### 3.3. Patients’ Adherence

Table 3 summarizes patient adherence based on survey responses. Of all respondents, 33 patients (25.38%) reported non-adherence to their prescribed treatment. Among them, 19 patients (57.58%) reported infrequent administration as the main reason, while 12 patients (36.36%) reported overdosing per administration. The primary reasons for non-adherence were bleeding (39.39%), tiredness from injections (36.36%), and lack of time (21.21%). Other adherence-related details, including injection administrator, mixed regimens and recent dose adjustments, are provided in Supplementary Table S3.

### 3.4. Annual Bleeding Events

The characteristics of ABEs are summarized in Table 4. A total of 396 bleeding events were recorded, with 69 events occurring in the prophylactic treatment group and 327 events in the non-prophylactic treatment group. Most episodes were moderate in severity, but their distribution differed significantly between groups (*p* < 0.001). Mild events accounted for 30.43% of bleeds in the prophylaxis group, compared to 6.12% in the non-prophylaxis group, while severe events remained uncommon in both groups (7.25% vs. 1.22%).

On a per-patient basis, the average ABEs were 1.47 in the prophylaxis group and 3.94 in the non-prophylaxis group. The distribution of ABEs differed significantly between the two groups (*p* < 0.001).

Adherence modified these effects. Among adherent patients, prophylaxis reduced ABEs by approximately three-fold (1.37 ± 3.04 vs. 3.64 ± 5.08; *p* < 0.001), whereas no significant difference was observed in the non-adherent patients.

**Figure 1:**
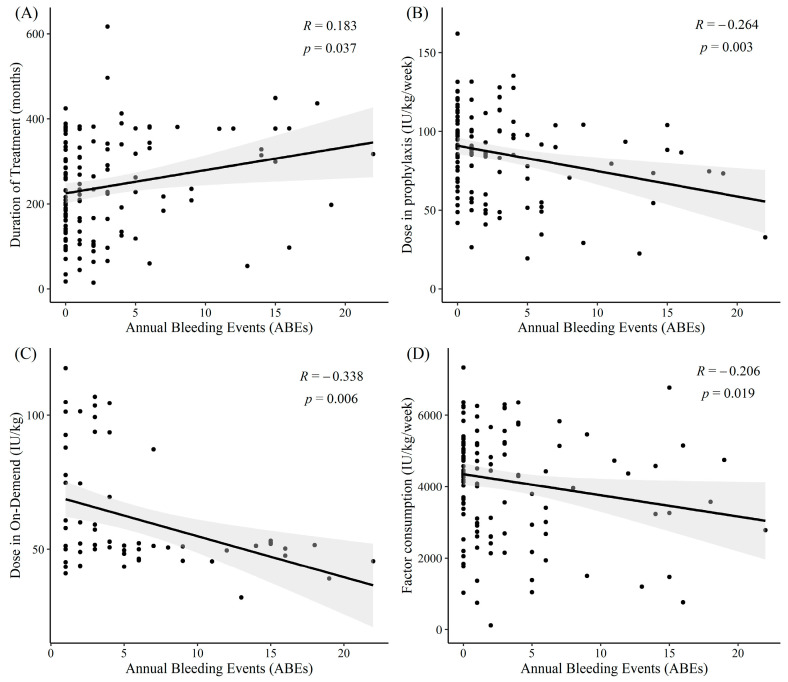
Correlations between annual bleeding events (ABEs) and treatment patterns. (**A**) Duration of treatment; (**B**) Dose in prophylaxis; (**C**) Dose in on-demand; (**D**) Factor consumption. Each subfigure shows a scatterplot with a least square line and its 95% confidence interval. ‘R’ represents the correlation coefficient and ‘*p*’ represents the *p*-value.

Figure 1 illustrates negative correlations between ABEs and prophylaxis/on-demand dosage and factor consumption, implying that higher doses are associated with lower bleeding events, regardless of the treatment pattern. Further details on the relationship between ABEs and demographics, clinical characteristics, treatment patterns, and patient adherence are presented in Supplementary Tables S4–S6.

### 3.5. Influence of Treatment Pattern and Patients’ Adherence on Annual Bleeding Events

**Figure 2:**
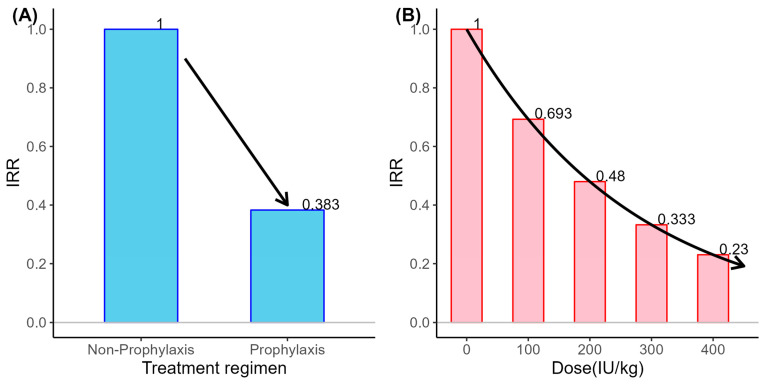
Relationships between treatment patterns and bleeding events. (**A**) Incidence rate ratio by treatment regimen for annual bleeding events. (**B**) Incidence rate ratio by the dose of factor IX for monthly bleeding events.

A univariate analysis was conducted using negative binomial regression to examine the factors influencing ABEs. The candidate covariates with *p*-values of less than 0.1 in the univariate (Supplementary Table S7), together with clinically relevant factors, were subsequently entered into the multiple analyses. In the multiple negative binomial regression (Table 5), prophylaxis remained the only independent predictor of annual ABEs, with an incidence rate ratio (IRR) of 0.383 (*p*-value = 0.011), corresponding to a 61.7% reduction in bleeding incidence. Figure 2A visually illustrates the effects of the treatment regimen on ABEs.

A second multiple model was fitted with a generalized linear mixed model (GLMM, negative binomial distribution) using monthly bleeding events data. The analysis revealed that only the dose per 100 IU/kg in the month of bleeding was significantly associated with bleeding events: each additional 100 IU/kg lowered the bleeding risk by 31% (IRR = 0.693, *p* < 0.001). Accordingly, a monthly dose of 400 IU/kg would be expected to reduce the incidence of bleeding events to about 23% (i.e., 0.693^4^) of that in a patient receiving no FIX during the same month (Figure 2B).

## 4. Discussion

In this study, we aimed to investigate treatment patterns and patient adherence in Korea among patients with haemophilia B, as well as to explore the relationships between various clinical factors and ABEs. Three principal findings emerged.

Firstly, prophylactic treatment was underutilized, with only 36.15% of patients meeting the criteria for sustained prophylaxis (≥90 IU/kg/week for ≥45 weeks), despite high reimbursement policies. Uptake was highest among adolescents and those with severe haemophilia, consistent with data from Ullman et al. [17]. However, the low rate in adults suggests that factors such as injection burden, work-life logistics, and limited patient education may act as persistent barriers in Korea.

Secondly, our model-based analyses revealed that prophylactic treatment significantly reduced annual bleeding events, and that the actual FIX dose in the month of bleeding was a stronger predictor of bleeding events than prophylaxis status alone. Each additional 100 IU/kg administered during the month of a bleed was associated with a 31% reduction in risk (IRR = 0.693, *p* < 0.001). This implies that adequate dosing intensity is essential, even among patients nominally categorized as on prophylaxis. Notably, over a half of severe cases in the non-prophylactic group received < 90 IU/kg/week, indicating a severity–dose mismatch requiring attention in clinical decision-making.

Thirdly, self-reported adherence in our cohort was sub-optimal: 25% of respondents acknowledged deviating from their prescribed regimen. The most frequent lapses were infusing less often than prescribed (58%) or administering an oversized single dose (36%). Notably, a breakthrough of bleeding—not the absence of symptoms reported in earlier studies [18]—was the main reason patients altered their schedule, followed by injection fatigue and lack of time. Non-adherence was nearly twice as common in the non-prophylactic treatment group (29%; 24/83) as in the prophylactic treatment group (19%; 9/47), suggesting that stable preventive schedules facilitate, but do not guarantee, compliance. Taken together, these findings point to reactive self-dosing behavior and highlight the need for continuous digital monitoring—such as smartphone infusion logs—to replace one-off recall surveys and enable timely clinical intervention.

International registries document a steady rise in high-dose prophylaxis, yet adherence to guideline intensity remains inconsistent [19,20]. National and international guidelines continue to prioritize routine prophylaxis for moderate to severe haemophilia B [21,22,23], and pharmacokinetic data show that FIX activity can be detected up to a week after administration when weekly exposure approaches or exceeds 100 IU/kg [15,24]. Real-world studies, such as the CHESSII [25,26] and a recent United States claims analysis confirm that bleeding may persist despite nominal prophylaxis, often due to under-dosing or suboptimal regimen rather than pharmacological failure. Our data provide an Asian perspective on the same issue: inadequate weekly exposure—especially in adults managed episodically—remains the dominant cause of residual bleeding in Korea. Personalized dose adjustment is therefore essential to balance cost, adherence, and bleed risk. Overall, our study reinforces current guideline recommendations for routine and adequately dosed prophylaxis but also argues for greater individualization. Population-pharmacokinetic tools, interval extension strategies, and shared decision-making could improve the adult uptake without inflating the overall factor consumption. From a health system perspective, monitoring actual FIX exposure—rather than relying solely on vial counts—could provide a clearer picture of treatment adequacy and help identify both under-dosing and overdosing.

Several limitations should temper the interpretation of these findings. Firstly, while treatment prescriptions and bleeding outcomes were monitored over a year, patients’ adherence was assessed only once through a one-month recall survey, creating a potential mismatch between the reported adherence and actual practice. Continuous digital monitoring would provide a more accurate picture. Secondly, the measurement of patients’ adherence relied on self-reported surveys, which may be influenced by an individual interpretation of questions and potential recall bias, particularly in cases where bleeding events occurred. Thirdly, brand-level information for both SHL and EHL FIX concentrates was not collected, precluding product-specific analyses.

## 5. Conclusions

This multi-center, real-world study shows that bleed prevention in Korean people with haemophilia B hinges on the actual amount of FIX infused. Our study established that sustained prophylaxis (≥90 IU/kg/week for ≥45 weeks) was achieved by only one-third of patients, and under-dosing was common even in severe haemophilia—particularly those in the non-prophylaxis cohort. Adequate weekly exposure lowered annualized bleeding events by about 62%, and each additional 100 IU/kg prescribed within a month further reduced the bleed risk by 31%. Despite Korea’s single-payer system, which minimizes direct treatment costs, one quarter of patients were non-adherent; this finding indicates that logistical and behavioral factors, rather than financial barriers, are the main obstacles to effective care. Tailored dose optimization and continuous, objective monitoring will therefore be essential to close this gap and further improve clinical outcomes.

Future work should: (i) integrate continuous digital tools (e.g., smartphone infusion logs, wearables) to capture real-time adherence and physical activity; (ii) evaluate extended-half-life, and non-factor and gene-therapy products within prospective national registries; and (iii) explore patient-reported barriers, particularly during the transition from adolescence to adulthood. A clear picture of delivered FIX exposure, day-to-day adherence, and emerging modalities will enable clinicians to fine-tune prophylaxis intensity while preserving health-system sustainability.

## Figures and Tables

**Table 1:** Baseline demographic and clinical characteristics of study participants, and treatment patterns during the 12-month observation period. Continuous variables are presented as mean ± standard deviation, and categorical variables as number (%).

Demographics and Clinical Characteristics		Treatment Patterns	
	Mean ± SD or *n* (%)		Mean ± SD or *n* (%)
Age (years)	31.56 ± 16.99	Treatment duration (months)	241.20 ± 116.43
Gender, *n* (%)		Half-life, *n* (%)	
Male	129 (99.23%)	Standard half-life	122 (93.85%)
Female	1 (0.77%)	Extended half-life	8 (6.15%)
Height (cm)	165.74 ± 16.79	Switch	0 (0.00%)
Weight (kg)	67.69 ± 19.43	Treatment regimen, *n* (%)	
BMI (kg/m^2^)	23.97 ± 4.33	Prophylaxis	47 (36.15%)
Disease duration (months)	294.22 ± 146.41	Non-prophylaxis	83 (63.85%)
Severity, *n* (%)		Prophylactic dose (IU/kg/week)	*n* = 126 86.26 ± 26.84
Severe	105 (80.77%)	Prophylaxis	*n* = 47 106.58 ± 13.42
Moderate	23 (17.69%)	Non-prophylaxis	*n* = 79 74.17 ± 25.52
Mild	2 (1.54%)	On-demand dose (IU/kg)	*n* = 64 61.27 ± 21.23
Factor IX activity level (%)	*n* = 109 9.13 ± 13.64	Prophylaxis	*n* = 14 69.97 ± 25.10
		Non-prophylaxis	*n* = 50 58.83 ± 19.61
		Factor consumption (IU/kg/week)	79.99 ± 29.10
		Prophylaxis	105.04 ± 11.60
		Non-prophylaxis	65.81 ± 26.34

Abbreviations: SD, standard deviation.

**Table 2:** Prophylaxis update and weekly FIX utilization. Continuous variables are presented with standard deviations, and categorical variables as frequencies with percentages.

**(A) Proportions of Patients Receiving Prophylaxis by Age and Severity**							
**Subgroup**		**Prophylaxis** **(*n* = 47)**			**Non-Prophylaxis** **(*n* = 83)**		
		***n* (%)**			***n* (%)**		
Age	0–11	6 (40%)			9 (60%)		
	12–17	9 (69.23%)			4 (30.77%)		
	≥18	32 (31.37%)			70 (68.63%)		
**(B) Weekly FIX Exposure**							
**Treatment Group**		**Prophylaxis**			**Non-Prophylaxis**		
			**Prophylactic Dose** **(IU/kg/week)**	**Factor Consumption** **(IU/kg/week)**		**Prophylactic Dose** **(IU/kg/week)**	**Factor consumption** **(IU/kg/week)**
		***n* (%)**	**Mean ± SD**	**Mean ± SD**	***n* (%)**	**Mean ± SD**	**Mean ± SD**
Mild		0 (0%)	-	-	2 (100%)	91.47 ± 48.18	80.03 ± 28.85
Moderate		4 (20%)	104.38 ± 16.10	100.17 ± 14.84	16 (80%)	72.40 ± 22.03	53.66 ± 31.41
Severe		43 (41.35%)	106.78 ± 13.35	105.49 ± 11.37	61 (58.65%)	74.06 ± 25.99	69.07 ± 23.76

Abbreviations: SD, standard deviation.

**Table 3:** Patients’ adherence based on patient survey. The patient survey was conducted once, and patients were asked about their experiences over the course of a month. Values are presented as frequencies and percentages.

Variables of Patients’ Adherence	
Adherence status, *n* (%)	
Adherent	97 (74.62%)
Non-adherent	33 (25.38%)
Number of non-adherents	4.00 ± 2.32
Types of non-adherence, *n* (%)	*n* = 33
Overdose injection per administration, *n* (%)	12 (36.36%)
Under-dose injection per administration, *n* (%)	5 (15.15%)
Over-frequent administration, *n* (%)	9 (27.27%)
Under-frequent administration, *n* (%)	19 (57.58%)
Reasons for non-adherence, *n* (%)	*n* = 33
Lack of time, *n* (%)	7 (21.21%)
Too frequent administration, *n* (%)	3 (9.09%)
Feel worsening of disease, *n* (%)	5 (15.15%)
Feel getting better of disease, *n* (%)	2 (6.06%)
Occurrence of bleeding, *n* (%)	13 (39.39%)
Too expensive, *n* (%)	1 (3.03%)
Forgetfulness, *n* (%)	4 (12.12%)
Lack of dosage, *n* (%)	0 (0.00%)
Tiredness from injections, *n* (%)	12 (36.36%)
Others, *n* (%)	5 (15.15%)

**Table 4:** Annual bleeding events. The means and standard deviations were calculated for continuous variables, while frequencies and percentages were calculated for categorical variables. *p*-values were calculated by comparing the prophylactic treatment and non-prophylactic treatment groups.

	Total (*n* = 130)	Prophylaxis (*n* = 47)	Non-Prophylaxis (*n* = 83)	*p*-Value
**(A) Bleeding event severity**				
Bleeding severity, cases (%)	396 (100.00%)	69 (100.00%)	327 (100.00%)	<0.001 ^†^
Mild	41 (10.35%)	21 (30.43%)	20 (6.12%)	
Moderate	346 (87.37%)	43 (62.32%)	303 (92.66%)	
Severe	9 (2.27%)	5 (7.25%)	4 (1.22%)	
**(B) ABEs per patient**				
Recorded bleeding events per patient during observation, Mean ± SD	3.05 ± 4.71	1.47 ± 2.91	3.94 ± 5.29	<0.001 ^§^
ABEs = 0, *n* (%)	52 (40.00%)	29 (61.70%)	23 (27.71%)	
ABEs > 0 and ≤3, *n* (%)	44 (33.85%)	12 (25.53%)	32 (38.55%)	
ABEs > 3 and ≤6, *n* (%)	16 (12.31%)	2 (4.26%)	14 (16.87%)	
ABEs > 6, *n* (%)	18 (13.85%)	4 (8.51%)	14 (16.87%)	
**(C) ABEs by adherence**				
ABEs according to patients’ adherence				0.308 ^‡^
Adherent	97 (74.62%) 2.75 ± 4.52	38 (80.85%) 1.37 ± 3.04	59 (71.08%) 3.64 ± 5.08	<0.001 ^§^
Non-adherent	33 (25.38%) 3.91 ± 5.22	9 (19.15%) 1.89 ± 2.37	24 (28.92%) 4.67 ± 5.81	0.211 ^§^

Abbreviations: SD, standard deviation; ^†^ *p*-value by Fisher’s exact test; ^‡^ *p*-value by chi-square test ^§^ *p*-value by Mann–Whitney *U* test.

**Table 5:** Independent predictors of bleeding events in two multiple models. Full coefficient lists—including non-significant covariates such as BMI, arthropathy, product half-life, and injection administrator—are provided in Supplementary Tables S8 and S9.

Predictors	Annual ABEs (Negative Binomial)		Monthly Bleeds (GLMM)	
	Exponentiated Estimates (95% CI)	*p*-Value	Exponentiated Estimates (95% CI)	*p*-Value
Treatment regimen				
Non-prophylaxis	ref			
Prophylaxis	0.3830 (0.182–0.805)	0.011	0.634 (0.315–1.275)	0.201
Dose (100 IU/kg) ^†^	**-**	**-**	0.693 (0.606–0.792)	<0.001
Patients’ adherence				
Non-adherent	ref			
Adherent	0.703 (0.383–1.292)	0.257	0.689 (0.361–1.313)	0.257
Age (years)	1.011 (0.989–1.033)	0.341	1.005 (0.981–1.030)	0.663

Abbreviations: CI, confidence interval. ^†^ Dose (100 IU/kg) was analyzed only in the GLMM (monthly data); no corresponding annual variable was entered in the negative binomial model.

## Data Availability

Restrictions apply to the availability of these data. Data were obtained from Pfizer and available from the authors with the permission of Pfizer.

## Supplementary Materials

The following are available online at https://www.mdpi.com/article/10.3390/jcm14134555/s1, Table S1: Patient characteristics based on medical chart review; Table S2: Detailed average dose and factor consumption; Table S3: Detailed adherence variables; Table S4: Comparisons of annual bleeding events (ABEs) by demographic and clinical characteristics; Table S5: Comparisons of annual bleeding events (ABEs) by treatment patterns; Table S6: Comparisons of annual bleeding events (ABEs) by patients’ adherence based on patient survey; Table S7: Univariate analysis of annual bleeding events (ABEs) with negative binomial regression; Table S8: Multiple analyses of annual bleeding events with negative binomial regression; Table S9: Multiple analyses of monthly bleeding events with generalized linear mixed model and a negative binomial distribution.
