# Supplementary material for: Characteristics and Treatment Patterns of Patients with Haemophilia B Receiving Recombinant Coagulation Factor IX
Source: J Clin Med. 2025 Jun 26;14(13):4555. doi: 10.3390/jcm14134555 (PMC12250204; doi:10.3390/jcm14134555)
Supplement: Supplementary file 1 [file jcm-14-04555-s001.zip › jcm-3633915-supplementary.pdf]

Web-Based Supplementary Materials for 'Characteristics and Treatment Patterns of Patients with Haemophilia B Receiving Recombinant Coagulation Factor IX'

Young Shil Park[1], Tai Ju Hwang[2], Sang Kyu Park[3], Eun Jin Choi[4], Jeong A Park[5], Hee Jo Baek[6], Chuhl Joo Lyu[7], Jae Hee Lee[8], Mi Kyung Kim[9], Ji Yoon Kim[10], Sun Ah Lee[11], Boram Park[12], Da-Hye Kim[12], Sung Beom Chung[12], Chung-Mo Nam[13], Yaeji Lee[14], Ki Young Yoo[15*]

[1] Department of Pediatrics, Kyung Hee University Hospital at Gangdong, Seoul, South Korea

[2] Korea Hemophilia Foundation Clinic, Gwangju, South Korea

[3] Korea Hemophilia Foundation Clinic, Busan, South Korea

[4] Department of Pediatrics, Daegu Catholic University Medical Center, Daegu, South Korea

[5] Department of Pediatrics, Inha University Hospital, Incheon, South Korea

[6] Department of Pediatrics, Chonnam National University Hwasun Hospital, Chonnam National University Medical School

[7] Department of Pediatric Hematology Oncology, Yonsei University College of Medicine, Seoul, South Korea

[8] Department of Pediatrics, Chungbuk National University School of Medicine, Chungbuk National University Hospital, Cheongju 28644, South Korea

[9] Department of Pediatrics, Presbyterian Medical Center, 365 Seowon-ro, Wansan-gu, Jeonju 54987, South Korea

[10] Department of Pediatrics, School of Medicine, Kyungpook National University, Kyungpook National University Hospital, Daegu, Daegu, South Korea

[11] Department of Internal Medicine, Daegu Fatima Hospital, Daegu, South Korea

[12] Medical Department, Pfizer Pharmaceuticals Korea Ltd, Seoul, South Korea

[13]Department of Preventive Medicine, Yonsei University College of Medicine, Seoul, South Korea

[14] Department of Biostatistics and Computing, Yonsei University, Seoul, South Korea

[15] Korea Hemophilia Foundation Clinic, Seoul, South Korea

Table S1. Patient characteristics based on medical chart review.

Demographics and Clinical Characteristics		Treatment Patterns	
**Inhibitors, *n* (%)**		**Prescribed weekly infusion for prophylaxis, cases (%)[#]**	642 (100.00%)
Yes	0 (0.00%)	≤1/week	174 (27.10%)
Transient	0 (0.00%)	1–2/week	287 (44.70%)
No	129 (99.23%)	2–3/week	136 (21.18%)
Unknown	1 (0.77%)	3–4/week	23 (3.58%)
**Arthropathy, *n* (%)**		> 4/week	22 (3.43%)
Yes	72 (55.38%)	**Changes of prophylactic dose, *n* (%)**	*n* = 126
No	58 (44.62%)	Yes	99 (78.57%)
**Arthropathy site, *n* (%)**		No	27 (21.43%)
Knee joints, *n* (%) Yes	36 (27.69%)	**Reasons for changing in prophylaxis, cases (%)[#]**	*n* = 572
No	94 (72.31%)	Lack of preventive effect	17 (2.97%)
Ankle joints, *n* (%) Yes	58 (44.62%)	Increased activity	0 (0.00%)
No	72 (55.38%)	Acute stage after surgery	4 (0.70%)
Elbow joints, *n* (%) Yes	34 (26.15%)	Subacute stage after surgery	23 (4.02%)
No	96 (73.85%)	Salivitis	0 (0.00%)
Shoulder joints, *n* (%) Yes	4 (3.08%)	Acute phase after severe bleeding	0 (0.00%)
No	126 (96.92%)	Others	518 (90.56%)
Hip joints, *n* (%) Yes	10 (7.69%)	Unknown	14 (2.45%)
No	120 (92.31%)		
Other joints, *n* (%) Yes	2 (1.54%)		
No	128 (98.46%)		
**HIV, *n* (%)**			
Positive	6 (4.62%)		
Negative	115 (88.46%)		
**Hepatitis B, *n* (%)**			
Acute	0 (0.00%)		
Chronic	3 (2.31%)		
Recovery after infection	3 (2.31%)		
No	108 (83.08%)		
Unknown	16 (12.31%)		

Table S2. Detailed Average dose and factor consumption.

	Mild (n = 2)	Moderate (n = 23)	Severe (n = 105)	P-Value	Extended Half-Life (n = 8)	Standard Half-Life (n = 75)	P-value‡
**(A) All**							
**Prophylactic dose (IU/kg/week)**				0.212			<0.001
n	2	20	104		8	118	
**Mean ± SD†**	91.47 ± 48.18	78.80 ± 24.42	87.59 ± 26.99		45.55 ± 10.18	89.02 ± 25.34	
**Factor consumption (IU/kg/week)**				0.013			<0.001
n	2	23	105		8	122	
**Mean ± SD†**	80.03 ± 28.85	61.75 ± 34.09	83.99 ± 26.59		45.92 ± 11.20	82.23 ± 28.53	
***(B)* Prophylactic Treatment**							
	**Mild (n = 0)**	**Moderate (n = 4)**	**Severe (n = 43)**	**P-Value**	**Extended Half-Life (n = 0)**	**Standard Half-Life (n = 47)**	**P-Value ‡**
**Prophylactic dose (IU/kg/week)**				0.619			-
n	0	4	43		0	47	
**Mean ± SD†**	-	104.38 ± 16.10	106.78 ± 13.35		-	106.58 ± 13.42	
**Factor consumption (IU/kg/week)**				0.38			-
n	0	4	43		0	47	
**Mean ± SD†**	-	100.17 ± 14.84	105.49 ± 11.37		-	105.04 ± 11.60	
***(C)* Non-prophylactic treatment**							
	**Mild (n = 2)**	**Moderate (n = 19)**	**Severe (n = 62)**	**P-value**	**Extended half-life (n = 8)**	**Standard half-life (n = 75)**	**P-value†**
**Prophylactic dose (IU/kg/week)**				0.753			<0.001
n	2	16	61		8	71	
**Mean ± SD**	91.47 ± 48.18	72.40 ± 22.03	74.06 ± 25.99		45.55 ± 10.18	77.39 ± 24.72	
**Factor consumption (IU/kg/week)**				0.097			0.01
n	2	19	62		8	75	
**Mean ± SD†**	80.03 ± 28.85	53.66 ± 31.41	69.07 ± 23.76		45.92 ± 11.20	67.93 ± 26.64	

Abbreviations: SD, standard deviation. † P-values are calculated using the Mann–Whitney *U* test.

Table S3. Detailed Adherence variables The patient survey was conducted once, and patients were asked about their experiences over the course of a month. The means and standard deviations were calculated for continuous variables, while frequencies and percentages were calculated for categorical variables.

Variables of Patients' Adherence		
**Injection administrator, *n* (%)**		
	Patient	80 (61.54%)
	Protector	33 (25.38%)
	Physician	17 (13.08%)
**Prescribed treatment regimen, *n* (%)**		
	Prophylaxis	79 (60.77%)
	On-demand 1†	10 (7.69%)
	On-demand 2†	5 (3.85%)
	Prophylaxis and On-demand 1	28 (21.54%)
	Prophylaxis and On-demand 2	6 (4.62%)
	Others	2 (1.54%)
**Treatment regimen switch, *n* (%)**		
	Yes	7 (5.38%)
	No	123 (94.62%)
**Switch type, *n* (%)**		*n* = 7
	On-demand to prophylaxis	4 (57.14%)
	Prophylaxis to prophylaxis and on-demand	1 (14.29%)
	Others	2 (28.57%)
**Bleeding in the past month, *n* (%)**		
	Yes	49 (37.69%)
	Total dose for a month before bleeding (IU/month)	20,046.06 ± 11,024.13
	Total number of doses for a month before bleeding (n/month)	5.41 ± 3.20
	No	81 (62.31%)
	Total dose for the past month (IU/month)	20,991.26 ± 9,393.71
	Total number of doses for the past month (n/month)	5.48 ± 2.26

† On-demand 1 represents the on-demand regimen after bleeding, while On-demand 2 represents the on-demand regimen before bleeding.

Table S4. Comparisons of annual bleeding events (ABEs) by demographic and clinical characteristics Mean ± standard deviation represents the mean of ABEs and their standard deviations for each characteristic. The correlation coefficients were calculated with a *p*-value for continuous characteristics, while the p-value was calculated using the methods described below for categorical characteristics.

Characteristics		*n* (%)	Mean ± SD	Correlation coefficients	*P*-value
**Age (years)**		130 (100.00%)		0.110	0.213†
**Gender, *n* (%)**					-
	Male	129 (99.23%)			
	Female	1 (0.77%)	3.06 ± 4.73		
**Height (cm)**		130 (100.00%)	1.00 ± NA	−0.015	0.867†
**Weight (kg)**		130 (100.00%)		0.031	0.730†
**BMI (kg/m²)**		130 (100.00%)		0.029	0.740†
**Disease duration (months)**		117 (90.00%)		0.123	0.185†
**Severity, *n* (%)**					
	Severe	105 (80.15%)			0.636‡
	Moderate	23 (17.56%)			
	Mild	3 (2.29%)			
**Inhibitors, *n* (%)**					0.275‡
	Yes	0 (0.00%)			
	High	0 (0.00%)			
	Low	0 (0.00%)	3.08 ± 4.64		
	Transient	0 (0.00%)	3.13 ± 5.28		
	No	129 (99.23%)	0.50 ± 0.71		
	Unknown	1 (0.77%)			
**Arthropathy, *n* (%)**					0.096§
	Yes	72 (55.38%)	-		
	No	58 (44.62%)	-		
	Unknown	0 (0.00%)	-		
**Arthropathy site, *n* (%)**			-		
Knee joints, *n* (%)			3.03 ± 4.73		0.080§
	Yes	36 (27.69%)	5.00 ± NA		
	No	94 (72.31%)			
Ankle joints, *n* (%)					0.222§
	Yes	58 (44.62%)	3.68 ± 5.16		
	No	72 (55.38%)	2.26 ± 4.00		
Elbow joints, *n* (%)			-		**0.011**§
	Yes	34 (26.15%)			
	No	96 (73.85%)			
Shoulder joints, *n* (%)					0.307§
	Yes	4 (3.08%)	3.75 ± 4.63		
	No	126 (96.92%)	2.78 ± 4.74		
Hip joints, *n* (%)					0.846§
	Yes	10 (7.69%)	3.41 ± 4.58		
	No	120 (92.31%)	2.75 ± 4.83		
Other joints, *n* (%)					0.083§

Yes	2 (1.54%)	4.47 ± 5.22		
No	128 (98.46%)	2.54 ± 4.44		
**HIV, *n* (%)**				0.481‡
Positive	6 (4.62%)	5.50 ± 6.66		
Negative	115 (88.46%)	2.97 ± 4.65		
Unknown	9 (6.92%)			
**Hepatitis B, *n* (%)**		3.60 ± 5.32		0.999‡
Chronic	3 (2.31%)	3.00 ± 4.68		
Recovery after infection	3 (2.31%)			
No	108 (83.08%)	11.00 ± 11.31		
Unknown	16 (12.31%)	2.92 ± 4.53		
**Factor IX activity level (%)**	109 (83.85%)		−0.139	0.151†

Abbreviations: SD, standard deviation

†P-value by Spearman's correlation analysis; ‡p-value by Kruskal–Wallis test; §p-value by Mann–Whitney *U* test

Table S5. Comparisons of annual bleeding events (ABEs) by treatment patterns

Mean ± standard deviation represents the mean of ABEs and their standard deviations for each characteristic. The correlation coefficients were calculated with a p-value for continuous characteristics, while the p-value was calculated using the methods described below for categorical characteristics.

Characteristics	*n* (%)	Mean ± SD	Correlation coefficients	*P*-value
**Treatment duration (months)**	130 (100.00%)		0.183	**0.037†**
**Half-life, *n* (%)**				0.269‡
SHL	122 (93.85%)	3.02 ± 4.76		
EHL	8 (6.15%)	3.50 ± 4.24		
Switch	0 (0.00%)	-		
SHL to EHL	0 (0.00%)	-		
EHL to SHL	0 (0.00%)	-		
SHL to EHL to SHL	0 (0.00%)	-		
EHL to SHL to EHL	0 (0.00%)	-		
**Treatment regimen, *n* (%)**				**<0.001‡**
Prophylaxis	47 (36.15%)	1.47 ± 2.91		
Non-prophylaxis	83 (63.85%)	3.94 ± 5.29		
**Prophylactic dose (IU/kg/week)**	*n* = 126		−0.264	**0.003†**
**On-demand dose (IU/kg)**	*n* = 64		−0.338	**0.006†**
**Change of dose in prophylaxis, *n* (%)**				0.528‡
Yes	99 (78.57%)	3.06 ± 4.70		
No	27 (21.43%)	2.19 ± 3.75		
**Factor consumption (IU/kg/week)**	130 (100.00%)		−0.206	**0.019†**
Prophylaxis	66 (50.77%)		−0.224	0.071†
On-demand 1§	3 (2.31%)		0.500	1.000†
On-demand 2§	1 (0.77%)		-	-
Prophylaxis and On-demand 1	60 (46.15%)		−0.086	0.511†
**Treatment duration (months)**	130 (100.00%)		0.183	**0.037†**

Abbreviations: SD, standard deviation; SHL, standard half-life; EHL, extended half-life

†*P*-value by Spearman's correlation analysis; ‡*p*-value by Mann–Whitney *U* test

§On-demand 1 represents the on-demand regimen after bleeding, while On-demand 2 represents the on-demand regimen before bleeding

Table S6. Comparisons of annual bleeding events (ABEs) by patients' adherence based on patient survey Mean ± standard deviation represents the mean of ABEs and their standard deviations for each characteristic. The correlation coefficients were calculated with a p-value for continuous characteristics, while the p-value was calculated using the methods described below for categorical characteristics.

Characteristics	*n* (%)	Mean ± SD	Correlation coefficients	*P*-value
**Injection administrator, *n* (%)**				0.449†
Patient	80 (61.54%)	3.69 ± 5.36		
Protector	33 (25.38%)	2.39 ± 3.83		
Physician	17 (13.08%)	1.29 ± 1.31		
**Prescribed treatment regimen, *n* (%)**				**0.001†**
Prophylaxis	79 (60.77%)	2.09 ± 3.86		
On-demand 1¶	10 (7.69%)	7.70 ± 7.51		
On-demand 2¶	5 (3.85%)	2.40 ± 1.67		
Prophylaxis and On-demand 1¶	28 (21.54%)	4.04 ± 5.01		
Prophylaxis and On-demand 2¶	6 (4.62%)	3.33 ± 5.85		
Others	2 (1.54%)	4.50 ± 3.54		
**Treatment regimen switch, *n* (%)**				0.237‡
Yes	7 (5.38%)	4.57 ± 6.58		
No	123 (94.62%)	2.96 ± 4.61		
**Switch type, *n* (%)**				0.619†
Prophylaxis to On-demand 1¶	0 (0.00%)	-		
Prophylaxis to On-demand 2¶	0 (0.00%)	-		
On-demand 1** to Prophylaxis	4 (57.14%)	6.75 ± 8.42		
On-demand 2** to Prophylaxis	0 (0.00%)	-		
Prophylaxis to Prophylaxis and On-demand	1 (14.29%)	1.00 ± NA		
On-demand to Prophylaxis and On-demand	0 (0.00%)	-		
Others	2 (28.57%)	2.00 ± 1.41		
**Bleeding in the past month, *n* (%)**				**<0.001‡**
Yes	49 (37.69%)	5.31 ± 5.96		
Total dose for a month before bleeding (IU/month)			–0.115	0.431§
Total number of doses for a month before bleeding (n/month)			–0.095	0.517§
No	81 (62.31%)	1.68 ± 3.09		
Total dose for the past month (IU/month)			–0.111	0.325§
Total number of doses for the past month (n/month)			–0.107	0.342§
**Adherence status, *n* (%)**				0.063‡
Adherent	97 (74.62%)	2.75 ± 4.52		
Non-adherent	33 (25.38%)	3.91 ± 5.22		
**Number of non-adherents**	33 (25.38%)		–0.126	0.486§
**Types of non-adherence, *n* (%)**	*n* = 33			
Overdose injection per administration				0.333‡
Yes	12 (36.36%)	5.17 ± 6.75		

No	21 (63.64%)	3.19 ± 4.13	
Under-dose injection per administration			0.959‡
Yes	5 (15.15%)	4.20 ± 6.22	
No	28 (84.85%)	3.86 ± 5.15	
Over-frequent administration			0.089‡
Yes	9 (27.27%)	7.44 ± 7.43	
No	24 (72.73%)	2.58 ± 3.46	
Under-frequent administration			0.237‡
Yes	19 (57.58%)	2.79 ± 3.82	
No	14 (42.42%)	5.43 ± 6.52	
**Reasons for non-adherence, *n* (%)**	*n* = 33		
Lack of time			0.517‡
Yes	7 (21.21%)	2.86 ± 3.58	
No	26 (78.79%)	4.19 ± 5.61	
Too frequent administration			0.504‡
Yes	3 (9.09%)	4.33 ± 4.16	
No	30 (90.91%)	3.87 ± 5.37	
Feel worsening of the disease			**0.009**‡
Yes	5 (15.15%)	11.00 ± 7.97	
No	28 (84.85%)	2.64 ± 3.46	
Feel getting better of the disease			**0.029**‡
Yes	2 (6.06%)	15.00 ± 0.00	
No	31 (93.94%)	3.19 ± 4.51	
Occurrence of bleeding			0.359‡
Yes	13 (39.39%)	5.46 ± 6.79	
No	20 (60.61%)	2.90 ± 3.75	
Too expensive			0.220‡
Yes	1 (3.03%)	9.00 ± NA	
No	32 (96.97%)	3.75 ± 5.22	
Forgetfulness			0.327‡
Yes	4 (12.12%)	1.00 ± 0.00	
No	29 (87.88%)	4.31 ± 5.46	
Lack of dosage			-
Yes	0 (0%)	-	
No	33 (100%)	3.91 ± 5.22	
Tiredness from injections			0.115‡
Yes	12 (36.36%)	2.08 ± 2.84	
No	21 (63.64%)	4.95 ± 6.00	
Others			0.475‡
Yes	5 (15.15%)	4.40 ± 4.16	
No	28 (84.85%)	3.82 ± 5.45	

†*P*-value by Kruskal–Wallis test; ‡*p*-value by Mann–Whitney *U* test; §*p*-value by Spearman's correlation analysis

¶On-demand 1 represents the on-demand regimen after bleeding, while On-demand 2 represents the on-demand regimen before bleeding

Table S7. Univariate analysis of annual bleeding events (ABEs) with negative binomial regression.

	Coefficients	S.E	Exponential estimates (95% CI)	*P*-value
**Treatment regimen**				
Non-prophylaxis	ref			
Prophylaxis	−0.987	0.292	0.373 (0.212–0.667)	**0.001**
**Patients' adherence**				
Non-adherent	ref			
Adherent	−0.351	0.321	0.704 (0.364–1.293)	0.274
**Age (years)**	0.015	0.008	1.015 (0.997–1.033)	**0.072**
**Gender**				
Female	ref			
Male	1.119	1.816	3.062 (0.012–106.336)	0.538
**Height (cm)**	0.003	0.009	1.003 (0.985–1.018)	0.752
**Weight (kg)**	0.003	0.007	1.003 (0.987–1.017)	0.724
**BMI (kg/m^2)**	0.010	0.033	1.011 (0.944–1.082)	0.750
**Disease duration (months)**	0.002	0.001	1.002 (1.000–1.004)	**0.064**
**Severity**				
Severe	ref			
Moderate	0.017	0.369	1.018 (0.511–2.206)	0.962
Mild	−1.817	1.467	0.163 (0.006–5.219)	0.216
**Arthropathy**				
Yes	ref			
No	−0.488	0.283	0.614 (0.353–1.075)	**0.085**
**Arthropathy site**				
Knee joints				
No	ref			
Yes	0.301	0.313	1.351 (0.744–2.561)	0.337
Ankle joints				
No	ref			
Yes	0.216	0.284	1.241 (0.712–2.182)	0.447
Elbow joints				
No	ref			

Yes	0.565	0.313	1.759 (0.971–3.341)	**0.071**
Shoulder joints				
No	ref			
Yes	0.617	0.796	1.853 (0.498–13.630)	0.438
Hip joints				
No	ref			
Yes	0.182	0.527	1.200 (0.470–3.901)	0.730
Other joints				
No	ref			
Yes	1.326	1.085	3.765 (0.724–85.193)	0.222
**HIV**				
Negative	ref			
Positive	0.892	0.645	2.439 (0.815–11.277)	0.167
Unknown	−0.073	0.557	0.929 (0.343–3.228)	0.895
**Hepatitis B**				
No	ref			
Chronic	−0.662	0.987	0.516 (0.092–6.118)	0.503
Recovery after infection	−0.662	0.987	0.516 (0.092–6.118)	0.503
Unknown	−0.335	0.438	0.716 (0.320–1.821)	0.445
**Factor IX activity level (%)**	−0.011	0.012	0.989 (0.969–1.015)	0.371
**Treatment duration (months)**	0.003	0.001	1.003 (1.000–1.005)	**0.024**
**Half-life**				
Standard half-life	ref			
Extended half-life	0.149	0.586	1.160 (0.415–4.416)	0.800
**Factor consumption (1,000 IU/kg/week)**	−0.879	0.479	0.415 (0.164–1.017)	**0.066**
**Injection administrator**				
Patient	ref			
Protector	−0.432	0.328	0.649 (0.347–1.266)	0.188
Physician	−1.047	0.449	0.351 (0.150–0.893)	**0.020**
**Treatment regimen switch**				
No	ref			
Yes	0.435	0.614	1.545 (0.534–6.442)	0.479
**Bleeding in the past month**				
No	ref			

Yes	1.151	0.267	3.160 (1.885–5.387)	**<0.001**

Abbreviations: S.E, standard error; CI, confidence interval

Table S8. Multiple analyses of annual bleeding events with negative binomial regression.

		Coefficients	S.E	Exponentiated Estimates (95% CI)	*P*-Value
**Treatment regimen**					
	Non-prophylaxis	ref			
	Prophylaxis	−0.9590	0.379	0.3830 (0.182–0.805)	**0.011**
**Patients' adherence**					
	Non-adherent	ref			
	Adherent	−0.352	0.310	0.703 (0.383–1.292)	0.257
**Age (years)**		0.011	0.011	1.011 (0.989–1.033)	0.341
**BMI (kg/m²)**		−0.039	0.037	0.961 (0.894–1.033)	0.285
**Severity**					
	Severe	ref			
	Moderate	−0.013	0.428	0.987 (0.426–2.284)	0.975
	Mild	−1.934	1.484	0.145 (0.008–2.650)	0.193
**Arthropathy**					
	Yes	ref			
	No	−0.513	0.346	0.599 (0.304–1.179)	0.138
**Treatment duration (months)**		0.001	0.002	1.001 (0.998–1.005)	0.542
**Half-life**					
	Standard half-life	ref			
	Extended half-life	0.336	0.634	1.400 (0.404–4.847)	0.596
**Factor consumption (1,000 IU/kg/week)**		−0.107	0.623	0.899 (0.265–3.051)	0.864
**Injection administrator**					
	Patient	Ref			
	Protector	0.096	0.401	1.101 (0.502–2.415)	0.810
	Physician	−0.325	0.470	0.722 (0.287–1.816)	0.489

Abbreviations: S.E, standard error; CI, confidence interval.

Table S9. Multiple analyses of monthly bleeding events with generalised linear mixed model and a negative binomial distribution. In this analysis, 'dose before' refers to the total prescribed dose in the previous month, while 'dose' refers to the total prescribed dose in the month in which the bleeding event occurred.

		Coefficients	S.E	Exponentiated Estimates (95% CI*)	*P*-Value
**Treatment regimen**					
	Non-prophylaxis	ref			
	Prophylaxis	−0.456	0.357	0.634 (0.315–1.275)	0.201
**Dose (100 IU/kg)**		−0.367	0.068	0.693 (0.606–0.792)	**<0.001**
**Dose before (100 IU/kg)**		−0.013	0.043	0.987 (0.906–1.074)	0.759
**Patients' adherence**					
	Non-adherent	ref			
	Adherent	−0.373	0.329	0.689 (0.361–1.313)	0.257
**Age (years)**		0.005	0.012	1.005 (0.981–1.030)	0.663
**BMI (kg/m^2)**		−0.023	0.039	0.977 (0.906–1.055)	0.560
**Severity, *n* (%)**					
	Severe	ref			
	Moderate	−0.201	0.446	0.818 (0.341–1.963)	0.653
	Mild	−1.503	1.581	0.222 (0.010–4.931)	0.342
**Arthropathy, *n* (%)**					
	Yes	ref			
	No	−0.489	0.381	0.613 (0.290–1.294)	0.199
**Treatment duration (months)**		0.002	0.002	1.002 (0.998–1.006)	0.333
**Half-life**					
	Standard half-life	ref			
	Extended half-life	0.193	0.638	1.213 (0.347–4.236)	0.763
**Injection administrator**					
	Patient	ref			
	Protector	0.210	0.430	1.233 (0.531–2.863)	0.625
	Physician	−0.224	0.501	0.800 (0.300–2.133)	0.655

Abbreviations: S.E, standard error; CI, confidence interval.
